# Cutting an NKG2D Ligand Short: Cellular Processing of the Peculiar Human NKG2D Ligand ULBP4

**DOI:** 10.3389/fimmu.2018.00620

**Published:** 2018-03-29

**Authors:** Tobias Zöller, Mareike Wittenbrink, Meike Hoffmeister, Alexander Steinle

**Affiliations:** ^1^Institute for Molecular Medicine, Goethe University Frankfurt am Main, Frankfurt am Main, Germany; ^2^Institute of Biochemistry II, Goethe University Frankfurt am Main, Frankfurt am Main, Germany; ^3^Brandenburg Medical School (MHB) Theodor Fontane, Institute of Biochemistry, Neuruppin, Germany

**Keywords:** NKG2D, ULBP4, NK cells, shedding, antibodies

## Abstract

Stress-induced cell surface expression of MHC class I-related glycoproteins of the MIC and ULBP families allows for immune recognition of dangerous “self cells” by human cytotoxic lymphocytes *via* the NKG2D receptor. With two MIC molecules (MICA and MICB) and six ULBP molecules (ULBP1–6), there are a total of eight human NKG2D ligands (NKG2DL). Since the discovery of the NKG2D–NKG2DL system, the cause for both redundancy and diversity of NKG2DL has been a major and ongoing matter of debate. NKG2DL diversity has been attributed, among others, to the selective pressure by viral immunoevasins, to diverse regulation of expression, to differential tissue expression as well as to variations in receptor interactions. Here, we critically review the current state of knowledge on the poorly studied human NKG2DL ULBP4. Summarizing available facts and previous studies, we picture ULBP4 as a peculiar ULBP family member distinct from other ULBP family members by various aspects. In addition, we provide novel experimental evidence suggesting that cellular processing gives rise to mature ULBP4 glycoproteins different to previous reports. Finally, we report on the proteolytic release of soluble ULBP4 and discuss these results in the light of known mechanisms for generation of soluble NKG2DL.

## Introduction

NKG2D is an activating, homodimeric C-type lectin-like immunoreceptor almost exclusively, but broadly, expressed on human cytotoxic lymphocytes endowing such killer cells with the capacity to detect and destroy dangerous “self cells” by means of the “induced-self” recognition mode (1–4). Upon NKG2D ligation, activating signals are intracellularly transduced *via* the NKG2D-associated adaptor protein DAP10 with subsequent activation of the phosphatidylinositol-3-kinase and the Grb2-Vav signaling pathways (5, 6). These signaling pathways stimulate cellular cytotoxicity, but also promote cytokine secretion by NK cells, CD8 αβ T cells, and γδ T cells (3, 7–9). NKG2D-mediated “induced-self” recognition is facilitated by various MHC class I-related cell surface glycoproteins, which usually are not or barely expressed on “healthy” cells but are strongly upregulated at the cell surface upon cellular stress, exposure to PAMPs, viral infection, or malignant transformation, thereby promoting cytolysis of “harmful” cells through engagement of NKG2D (1, 4, 10).

In humans, there are eight known ligands for NKG2D including the two MHC-encoded and MHC class I chain-related glycoproteins A and B (MICA and MICB) as well as the six non-MHC-encoded, UL16-binding proteins (ULBP1–6) (1, 4, 11, 12). MICA/B molecules are comprised of an MHC class-I-like α1α2 superdomain followed by an Ig-like α3 domain, a transmembrane domain, and a cytoplasmic domain (1, 4, 12, 13). By contrast, ULBP ectodomains comprise only the MHC class I-like α1α2 superdomain, which serves as NKG2D binding platform, and which is directly attached to the cellular membrane *via* a glycosylphosphatidylinositol (GPI) anchor (ULBP1, -2, -3, and -6) or followed by a transmembrane domain and a short (ULBP4) or long (ULBP5) cytoplasmic tail, respectively (11, 12, 14, 15). More recently, it has been shown that the common truncated MICA allelic variant MICA*08 can also be membrane attached *via* a GPI anchor (16).

**Figure 1:**
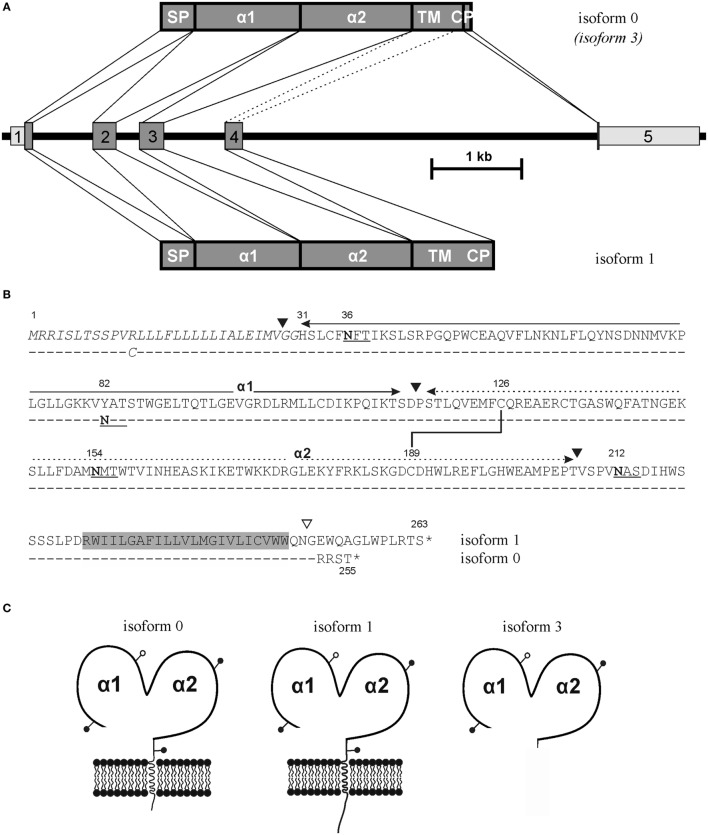
ULBP4 isoforms generated by differential splicing. **(A)** Differential usage of an internal splice site in exon 4 gives rise to the major full-length ULBP4 isoforms 0 and 1, respectively, while exclusion of exon 4 by alternative splicing (stippled lines) gives rise to the putatively secreted soluble ULBP4 isoform 3. Depicted is the exon–intron structure of the ULBP4/RAET1E gene and the alternatively spliced gene products representing isoforms 0, 3, and 1. **(B)** Amino acid sequence alignment of ULBP4 isoform 1 [upper; GenBank accession no. AY252119 (18)] and isoform 0 (lower; GenBank accession no. MH020173). Exon boundaries are indicated by black triangles except for the exon 4/5 boundary of isoform 0 (open triangle). N-linked glycosylation sites are underlined with asparagines in bold. Signal peptide sequence (Met 1 to Gly 30) is italicized, transmembrane domain highlighted in gray. Disulfide bond between Cys 126 and Cys 189 highly conserved in the MHC class I fold is indicated. **(C)** Scheme of ULBP4 isoforms 0, 1, and 3 with N-glycosylation sites indicated by pins.

ULBP4 is encoded at the centromeric end of the ULBP gene cluster on the long arm of human chromosome 6 by the *RAET1E* locus (11, 17). ULBP4 glycoproteins have first been described in 2003 by Cosman and colleagues (18) as well as by Coukos and colleagues (19). Both groups identified ULBP4 based on *in silico* screens of human genomic sequences searching for relatives of the ULBP family members ULBP1, ULBP2, and ULBP3, which had previously been discovered during a search for binding partners of the HCMV glycoprotein UL16 and been named accordingly (14). Of note, ULBP4, like ULBP3, is not bound by the HCMV glycoprotein UL16 (18, 20, 21) and therefore can be considered a misnomer. Both original studies (18, 19) described ULBP4 cDNA encoding for a polypeptide of 263 amino acids (including the signal peptide) and giving rise to a mature cell surface-bound protein of 235 amino acids (~27 kDa). This ULBP4 polypeptide is encoded by four exons with exon 1 encoding for the signal peptide, exon 2 for the α1 domain, exon 3 for the α2 domain, and exon 4 for the short serine-rich stalk, the transmembrane region, and a short cytoplasmic domain (Figure 1A). This originally reported ULBP4 variant has meanwhile been termed isoform 1 by the Uniprot database^1^ (22). Five additional ULBP4 isoforms (isoforms 2–6) are referenced in the Uniprot database originating from alternative splicing (23, 24) and will be discussed later. Sequence analyses and phylogenetic trees constructed from the comparison of ULBP α1α2 superdomains strongly suggest that ULBP4 has diverged from other primate ULBP molecules earliest and before the separation of Old and New World monkeys (25). In addition, ULBP4 is the most polymorphic member of the ULBP family of proteins (11, 26, 27), although there is no functional rationale for this polymorphism. There are some reports of ULBP4 being expressed by various tumors, EBV-infected B cells, and cytokine-activated NK cells that may be relevant for the NKG2D-mediated immunosurveillance and immunoregulation in these settings (28–33). In our approach to this peculiar NKG2DL, we realized that ULBP4 glycoproteins are poorly characterized and that there exist substantial inconsistencies when comparing the literature and publicly available databases on rather basic issues such as on biochemical properties and expression by cell lines and tissues. Hence, we set out to study expression and biochemical properties of ULBP4 molecules.

## Materials and Methods

### Cells

C1R and HepG2 cells were cultivated in RPMI 1640 (Sigma-Aldrich, St. Louis, MO, USA), HCT116 and HaCaT cells in Iscove’s Modified Dulbecco’s Medium (Sigma-Aldrich), and Hela cells in Dulbecco’s Modified Eagle Medium (Thermo Fisher Scientific, Waltham, MA, USA). All media were supplemented with 10% FCS (Biochrome, Berlin, Germany), 2 mM l-glutamine, 100 U/ml penicillin, 100 mg/ml streptomycin (both from Sigma-Aldrich), and RPMI 1640 in addition with 1 mM sodium pyruvate (Thermo Fisher). 293F cells were cultured in FreeStyle™ F17 Expression Medium (Thermo Fisher) with 0.1% Pluronic F-68 (Thermo Fisher), 100 U/ml penicillin, 100 mg/ml streptomycin, and 8 mM l-glutamine. C1R stably transfected with RSV.5neo containing the cDNA MICA*07, MICA*08 (21), ULBP4 (isoform 0), and ULBP4 (isoform 1) were cultivated in presence of 1.8 mg/ml G418.

### Antibodies

Anti-ULBP4 mAb clone 709116 and ULBP4-specific goat polyclonal antibodies (pAb) were from R&D (Minneapolis, MN, USA), anti-ULBP4 mAb clone 6E6 from Santa Cruz (Dallas, TX, USA) and the anti-hexahistidine-tag mAb His.H8 from Thermo Fisher. Secondary staining reagents allophycocyanin-conjugated goat anti-mouse Ig (GAM-APC), Alexa-Fluor-488-conjugated donkey anti-goat Ig (DAG-488), and phycoerythrin-conjugated streptavidin (SA-PE) were from Jackson ImmunoResearch (West Grove, PA, USA). MICA-specific mAb AMO1 and MICA/B-specific mAb BAMO3 were previously described (34). Anti-ULBP4 mAb DUMO1 was generated by immunizing BALB/c mice with P815-ULBP4 transfectants and soluble ULBP4 (sULBP4) by standard hybridoma technology as previously described (35) and isotyped as IgG1. Supernatants of hybridoma were screened for binding to 293-ULBP4 transfectants by flow cytometry. DUMO1 binds to P815, 293, and COS-7 cells transfected with ULBP4, but not to the respective mock transfectants, and DUMO1 does not cross-react with ULBP1, ULBP2, and ULBP3, respectively (data not shown).

### Flow Cytometry

Cells were harvested, washed twice with fluorescence-activated cell sorter (FACS) buffer (PBS, 2% FCS, 2 mM EDTA, and 0.01% sodium azide) and stained with 10 µg/ml of the primary antibody for 20 min at 4°C. Then, cells were washed again with FACS buffer and stained with DAG-488 (10 µg/ml) or GAM-APC (1.25 µg/ml) for 20 min at 4°C. After additional washing, flow cytometry analyses were performed using a FACS Canto II (BD Biosciences, Heidelberg, Germany) and data analyzed using FlowJo (Tree Star, Ashland, OH, USA). Biotinylated soluble truncated standard ULBP4 (ststULBP4) was immobilized on streptavidin-coated microspheres (Bangs Laboratories, Fishers, IN, USA) by incubating 5 µg microspheres with 5 µg/ml biotinylated ststULBP4 for 15 min at 4°C. Subsequently, ststULBP4-loaded microspheres were washed twice with FACS buffer, stained with antibodies, and analyzed by flow cytometry as described earlier. The specific fluorescence intensity was calculated by subtracting the median fluorescence intensity (MFI) of the isotype control from the MFI of the antibody of interest.

### Production of sULBP4

**Figure 2:**
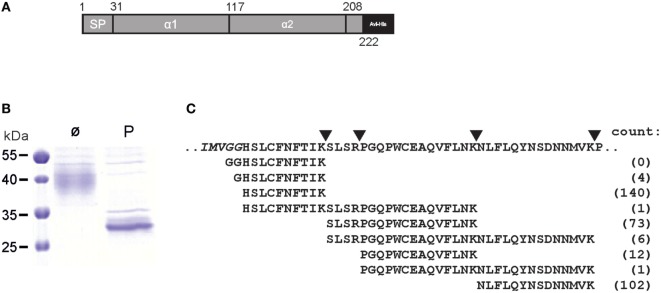
Aminoterminus of mature ULBP4 glycoproteins. **(A)** Scheme of soluble carboxyterminally truncated and tagged ULBP4 (stULBP4) used for mass spectrometric analyses. **(B)** Affinity-purified stULBP4 from supernatants of transfected 293F cells was treated either with PNGaseF (P) or left untreated (ø), separated by SDS-PAGE, and visualized by Coomassie staining. **(C)** Abundancy of aminoterminal stULBP4 fragments as determined by mass spectrometry. stULBP4 was digested with trypsin and LysC, and resulting fragments were analyzed and counted by mass spectrometry.

A cDNA encoding for the first 222 amino acids of ULBP4 (Met 1 to Ser 222) was cloned into the pFUSE vector (InvivoGen, San Diego, CA, USA) in front of an AviTag and a hexahistidine tag (Figure 2A) replacing the IL-2 signal sequence and the Fc-encoding region of the vector. The resulting plasmid encoding for soluble carboxyterminally truncated and tagged ULBP4 (stULBP4) was used for transient transfection of 293F cells. Supernatants were harvested 4 days later and stULBP4 affinity-purified using HisPurTM Ni-NTA spin columns (Thermo Scientific) according to the manufacturer’s protocol. Eluted stULBP4 was concentrated using Amicon^®^ Ultra Centrifugal Filter units (Merck, Darmstadt, Germany) and further purified by size exclusion chromatography using a Hiload™ 16/60 Superdex™ 200 column (GE Healthcare, Chicago, IL, USA) on an ÄKTA purifier (GE Healthcare). stULBP4 was collected and analyzed by SDS-PAGE (Figure 2B). For determining concentrations of sULBP4 and sMICA in culture supernatants, ststULBP4 (His 31 to Ser 222 followed by an AviTag and a hexahistidine tag) or soluble truncated standard MICA07 (ststMICA) (Glu 24 to Gln 304 followed by an AviTag and a hexahistidine tag) was produced as described above for stULBP4 in 293F cells and purified by Ni-NTA spin columns except that the vector-encoded IL-2 signal sequence was placed in front of the coding regions of mature ULBP4 and MICA glycoproteins. Biotinylation of ststULBP4 was done using BirA Biotin-protein ligase (Avidity, Aurora, CO, USA) according to the manufacturer’s protocol.

### Quantitative Real-Time PCR

Cellular RNA was isolated using pegGOLD TriFast™ (Peqlab, Erlangen, Germany) according to the manufacturer’s protocol. RNA from human esophagus and skin was purchased from Thermo Fisher. RNA was treated with RNAse-free DNAse (Promega, Madison, WI, USA) and subsequently converted into cDNA using M-MLV RT RNAse (H−) Point Mutant (Promega) according to the manufacturer’s protocol. Quantitative PCR (qPCR) was performed using SYBR Green technology (Roche, Basel, Switzerland) on a StepOnePlus Real-Time PCR System (Applied Biosystems, Foster City, CA, USA). For amplification of ULBP4, oligonucleotides Ex3fw (5′-CTGGCTCAGGGAATTCTTAGG-3′) and Ex4rv (5′-CTAGAAGAAGACCAGTGGATATC-3′) were used. To selectively amplify ULBP4 isoforms 0 or 1, the forward primer ULBP4_fw (5′-TACCAGATAGATGGATCATCCTG-3′) was combined with the reverse primers ULBP4_Iso0_rv (5′-CTAGGTGGATCTTCTGCCATT-3′) or ULBP4_Iso1_rv (5′-CTAAGACGTCCTCAAGGGCC-3′), respectively (all from Sigma-Aldrich). Copy numbers were normalized with the ΔΔCt method using 18S rRNA as previously described (35).

### Immunoblotting

HeLa cells were harvested, washed twice with PBS and resuspended with ice-cold NP40-Lysis-Puffer [1% NP40, 50 mM Tris, 150 mM NaCl, Complete Protease Inhibitor (Roche, Mannheim, Germany)]. Lysates were incubated for 20 min on ice and then centrifuged for 15 min at 17,000 *g*. ULBP4 was immunoprecipitated with biotinylated DUMO1 coupled to streptavidin magnetic beads (Thermo Fisher Scientific) for 3 h at 4°C. Immunoprecipitates were washed several times and eluted using denaturation buffer [0.5% SDS, 40 mM dithiothreitol (DTT)] for 5 min at 95°C. Deglycosylation was performed using endoglycosidase H or peptide-N-glycosidase F (both from New England Biolabs, Ipswich, MA, USA) according to the manufacturer’s protocols. Immunoprecipitates were separated using SDS-PAGE and blotted onto a PVDF membrane (Carl Roth, Karlsruhe, Germany). Membrane was blocked using 5% non-fat dried milk powder (AppliChem) in TBST (150 mM NaCl, 10 mM Tris, 0.1% Tween 20) and then probed with 2 µg/ml ULBP4-specific pAb, and subsequently with 0.16 µg/ml HRP-conjugated donkey anti-goat-Ig (Santa Cruz). Immunoblots were developed with HRP-Juice Plus (PJK, Kleinbittersdorf, Germany) using a Fusion SL imaging system (Vilber Lourmat).

### Detection of sULBP4 and sMICA

For detection of sULBP4 and sMICA, anti-ULBP4 pAb (R&D Systems) or mAb AMO1 were immobilized on MagPlex-C microspheres (Luminex, Austin, TX, USA), respectively, according to the manufacturer’s instructions. Cell culture supernatants were diluted 1:1 in PBS with 1% BSA (dilution buffer) and added together with SA-PE and either biotinylated DUMO1 (for sULBP4 detection) or biotinylated BAMO3 (for sMICA detection) to 500 microspheres. Standard curves were generated by titrating ststULBP4 or ststMICA. Samples were incubated overnight at room temperature, washed twice in dilution buffer, and measured on a Luminex 100 system (Luminex). All samples were measured in triplicates.

### In-Solution Digestion

Purified stULBP4 protein samples were precipitated with acetone, and subsequently in-solution digestion was performed as described (36). In brief, protein pellets were washed and resuspended in denaturation buffer containing 6 M urea and 2 M thiourea. Proteins were reduced with 1 mM DTT, alkylated with 5.5 mM iodoacetamide and digested with the endopeptidase LysC (Wako) for 3 h and sequencing-grade trypsin (Promega) overnight. Peptide mixtures were concentrated and desalted using the Stop and Go Extraction technique (37).

### Liquid Chromatography and Mass Spectrometry

A binary buffer system consisting of buffer A (0.1% formic acid) and buffer B (80% acetonitrile, 0.1% formic acid) was used for peptide separation on an Easy-nLC 1200 (Thermo Fisher Scientific). This system was coupled *via* a nano electrospray ionization source to the quadrupole-based Q Exactive HF benchtop mass spectrometer (38). Peptide elution from the in-house packed 18 cm (1.9 µm C18 Beads, Dr. Maisch Germany) column was achieved by increasing the relative amount of B from 10 to 38% in a linear gradient within 23 min at a column temperature of 40°C. Followed by an increase to 100% B within 7 min and gradients were completed by a re-equilibration to 5% B.Q Exactive HF settings: MS spectra were acquired using 3E6 as an AGC target, a maximal injection time of 20 ms and a 60,000 resolution at 300 *m*/*z*. The mass spectrometer operated in a data-dependent Top15 mode with subsequent acquisition of higher-energy collisional dissociation fragmentation MS/MS spectra of the top 15 most intense peaks. Resolution for MS/MS spectra was set to 15,000 at 200 *m*/*z*, AGC target to 1E5, maximal injection time to 25 ms and the isolation window to 1.6 Th.

### Mass Spectrometry Data Processing and Analysis

All acquired raw files were processed using MaxQuant (1.5.3.30) (39) and the implemented Andromeda search engine (40). For protein assignment, electrospray ionization-tandem mass spectrometry (ESI-MS/MS) fragmentation spectra were correlated with the Uniprot human database (v. 2017) with manually added peptide sequences of ULBP4 starting with all possible N-termini of the mature protein. Searches were performed with tryptic specifications and default settings for mass tolerances for MS and MS/MS spectra. Carbamidomethyl at cysteine residues was set as a fixed modification, while oxidation at methionine, acetylation at the N-terminus, and conversion from Asn to Asp were defined as variable modifications. The minimal peptide length was set to seven amino acids, and the false discovery rate for proteins and peptide spectrum matches to 1%. The match-between-run feature was used with a time window of 0.7 min.

### Statistics

Statistical analyses as detailed in the figure legends were performed using Prism 7 (GraphPad, San Diego, CA, USA).

## Results

### Aminoterminus of Mature ULBP4 Glycoproteins Is Recessed

We noted conflicting predictions for the aminoterminus of mature ULBP4 glycoproteins. In the original reports, the aminoterminus of mature ULBP4 glycoproteins, as generated by cleavage of the putative aminoterminal signal peptide, has been assigned in the absence of experimental evidence to glycine 29 along the exon 1/exon 2 boundaries (18, 19) presumably based on sequence alignments with other ULBP and MHC class I-related molecules (Figures 1A,B). However, we noted that the database Uniprot (22), in contrast to the existing literature, had assigned the aminoterminus of mature ULBP4 molecules to histidine 31 based on “manual assertion according to rules” (see text footnote 1). To clarify these conflicting predictions, we addressed this issue experimentally: soluble, carboxyterminally truncated and tagged ULBP4 ectodomains encompassing the α1, the α2 domain, and most of the serine-rich region, preceded by the “natural” ULBP4 signal peptide (stULBP4; Met 1 through Ser 222) were ectopically expressed in 293F cells, purified from the supernatants by Nickel-NTA spin columns (Figures 2A,B), fragmented by digestion with trypsin and LysC, and resulting stULBP4 fragments were subjected to liquid chromatography–mass spectrometry (LC–MS). Mass spectrometry identified all expected tryptic fragments almost completely covering the mature stULBP4 polypeptide (data not shown). The aminoterminus of mature ULBP4 molecules, however, is generated by cleavage of the signal peptide during cotranslational translocation into the ER, and, accordingly, potentially resulting in the peptides GGHSLCFNFTIK, GHSLCFNFTIK, or HSLCFNFTIK, respectively, depending on the actual cleavage site. While the fragment HSLCFNFTIK was detected at high abundance, the peptides GGHSLCFNFTIK and GHSLCFNFTIK were not or only very rarely detected (Figure 2C). These data strongly suggest that mature ULBP4 glycoproteins start with histidine 31 in line with the prediction by Uniprot and not as previously assumed with glycine 29. It remains to be determined how such a recessed β1 strand of the ULBP4 α1 domain may impact ULBP4 folding and function differently as compared with other ULBP molecules. Our mass spectrometric analyses also showed that the three putative N-glycosylation sites predicted by Uniprot at Asn 36 (α1 domain), Asn 154 (α2 domain), and Asn 212 (serine-rich region) all were glycosylated. In addition, we also detected N-linked glycosylation at Asn 82 (Figures 1B,C). Position 82 is polymorphic and occupied either by asparagine (stULBP4) or tyrosine (Uniprot reference sequence at http://www.uniprot.org/uniprot/Q8TD07), and therefore ULBP4 glycosylation can be expected to be variable within the human population with potential consequences for expression and detection. While Asn 82, Asn 154, and Asn 212 were glycosylated in almost all (>99%) tryptic fragments analyzed, glycosylation efficiency at Asn 36 appeared slightly reduced with ~90% of the fragments glycosylated (data not shown).

### Carboxyterminus of ULBP4 Is Variable due to Alternative Splicing

Original studies of ULBP4 reported that exon 4, in addition to the serine-rich stalk region, entirely encodes for both transmembrane and cytoplasmic ULBP4 residues (18, 19) (Figure 1). In the course of our studies on ULBP4, we characterized a commercially available EST clone (clone 601078687F1) derived from a cervical carcinoma cell line encoding for a ULBP4 variant with a divergent carboxyterminus (GenBank accession no. MH020173). This ULBP4 variant differs from the originally published sequence by an alternate and shorter cytoplasmic tail which is created by alternative splicing due to an alternative splice donor site within exon 4 (Figure 1). Accordingly, the 5′ portion of exon 4 is merged in frame with an additional exon located ~4 kb downstream (exon 5) which encodes the four carboxyterminal amino acids followed by a long 3′ UTR (Figure 1). Consequently, such ULBP4 isoforms have a distinct cytoplasmic domain shortened by eight amino acids as compared with the originally reported ULBP4 isoform 1 (Figure 1) (18, 19). In addition, Uniprot lists five other ULBP4 isoforms (isoforms 2–6) based on reports of alternatively spliced transcripts for which experimental evidence of protein expression is lacking or scarce (see text footnote 1). Among these, isoform 3 is the only isoform that also includes exon 5 as reported here for the new ULBP4 isoform. Isoform 3 was identified in the course of a broad screen by the secreted protein discovery initiative (24) that used computational and experimental approaches to identify human cDNA clones encoding for putatively secreted proteins preceded by a signal peptide. Of note, the cDNA of isoform 3 is composed of exons 1, 2, 3, and 5, but lacks exon 4 which encodes for the transmembrane region. Hence, isoform 3 corresponds to an alternatively spliced and potentially soluble isoform of the new full-length ULBP4 isoform reported here to which we refer as isoform 0 in the following (Figure 1). The original studies have shown that ULBP4 isoform 1 can be expressed at the cell surface of transfected cell lines and is functionally recognized by NK cells (18, 19). To assess whether the newly reported isoform 0 likewise gives rise to a ULBP4 glycoprotein that can be functionally expressed at the cell surface and consequently be recognized *via* NKG2D by NK cells and T cells, we generated a ULBP4-specific mAb.

#### ULBP4 Antibodies

**Figure 3:**
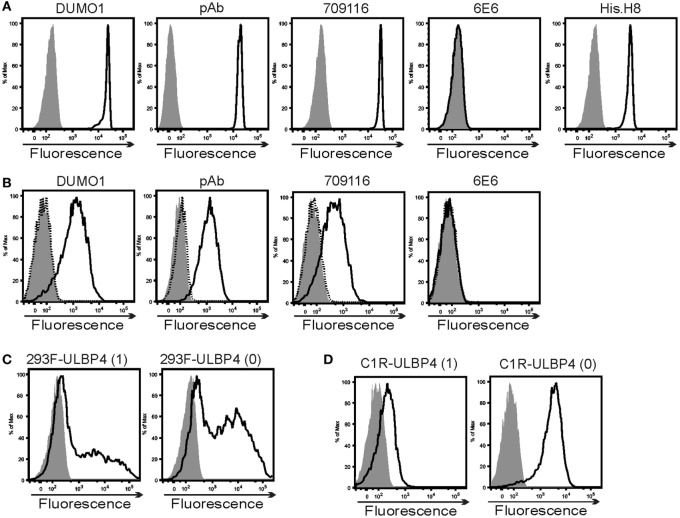
Characterization of ULBP4-specific antibodies. **(A)** Binding of various ULBP4 antibodies or anti-His-tag mAb (solid lines) to biotinylated ststULBP4 immobilized on streptavidin-coated microspheres. Control stainings are shaded. **(B)** Binding of various ULBP4 antibodies to C1R-ULBP4 isoform 0 cells (solid lines). Control stainings are shaded. No binding of ULBP4 antibodies to C1R-mock transfectants was detected (dotted). **(C,D)** Binding of DUMO1 to **(C)** 293F or **(D)** C1R cells transfected with ULBP4 isoform 1 or ULBP4 isoform 0. Control stainings are shaded.

The ULBP4-specific mAb DUMO1 was generated by immunizing mice with mouse mastocytoma cells P815 stably transfected with ULBP4. DUMO1 binds to ststULBP4 immobilized on microspheres (ststULBP4_im), but not to control microspheres (Figure 3A). DUMO1 also bound to 293F cells and C1R cells transfected with either ULBP4 isoform 0 or isoform 1, but not to the corresponding mock transfectants (Figures 3C,D). Apart from corroborating specificity of DUMO1 for cellular ULBP4, these data also demonstrate that ULBP4 isoform 0 can be broadly expressed at the cell surface. While our data also indicate that ULBP4 isoform 0 may allow for a brighter surface expression than ULBP4 isoform 1, this possibility needs to be validated by further experiments. For control, we included the few commercially available antibodies said to be ULBP4-specific. While ULBP4-specific pAb and mAb 709116 bound ststULBP4_im and ULBP4 transfectants similarly to DUMO1, we did not detect binding of mAb 6E6 neither to ststULBP_im nor to the ULBP4 transfectants by flow cytometry (Figures 3A,B), although mAb 6E6 brightly detected denatured ststULBP4 in immunoblotting (data not shown). mAb 6E6 has previously been used to demonstrate ULBP4 surface expression on EBV-infected cells and on placental exosomes (31, 41) as well as on ULBP4 transfectants^2^ which is puzzling in the light of our results.

#### ULBP4 Expression

**Figure 4:**
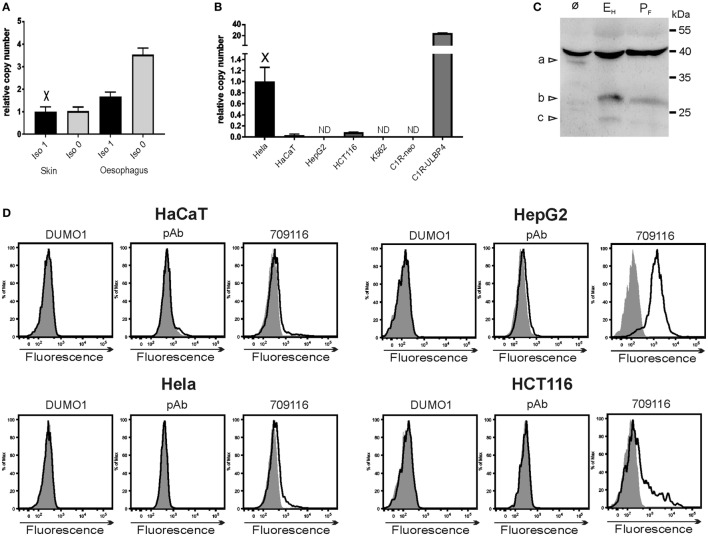
Detection of ULBP4 expression. **(A)** Abundance of ULBP4 isoforms 0 and 1 in human skin or esophagus as determined by quantitative PCR (qPCR). **(B)** Abundance of ULBP4 transcripts in various human cell lines as determined by qPCR. Abbreviation: ND, not detectable. **(C)** ULBP4 glycoproteins in lysates of HeLa cells were detected after immunoprecipitation with mAb DUMO1 with ULBP4-specific pAb in immunoblotting. Lysates were treated with endoglycosidase H (E_H_) or PNGase F (P_F_) before SDS-PAGE where indicated. Triangles indicate positions of glycosylated ULBP4 [a] in untreated lysates, and putative deglycosylated isoforms 0/1 [b] or 3 [c] in lysates treated with endoglycosidase H and PNGase F. **(D)** Binding of ULBP4 antibodies (solid lines) to various human cell lines. Control stainings are shaded.

Among human tissues, we found ULBP4 transcripts by qPCR most abundantly in tissues which are of ectodermal origin such as skin, esophagus, and cervix (data not shown). A preferential ULBP4 expression in human skin has already been reported by Cosman and colleagues (18) and a strong expression bias toward tissues of ectodermal origin is also documented by publicly available databases.^3^ We wondered whether isoforms 0 and 1 may be differentially expressed in such tissues but detected both at a comparable abundance in both skin and esophagus (Figure 4A). Assessing abundance of ULBP4 transcripts in a broad variety of human tumor cell lines, we detected ULBP4 transcripts most abundantly in the cervix carcinoma cell line HeLa, whereas ULBP4 transcripts were undetectable in liver cancer cells HepG2 or in the erythroleukemia line K562 (Figure 4B and data not shown) well in line with publicly available data sets.^4^ Of note, pronounced ULBP4 surface expression has been claimed based on binding of mAb 709116 for HepG2 cells by the supplier,^5^ for the erythroleukemia line K562 (42) and for cytokine-activated NK cells (32). By using DUMO1 and ULBP4-specific pAb, we were unable to detect ULBP4 surface expression on HeLa, HepG2, HCT116, and HaCat cells, respectively. By contrast, mAb 709116 bound to a substantial portion of HCT116 cells and brightly stained HepG2 cells (Figure 4D) as reported by the supplier and as previously reported for another not publicly available mAb (29), although we were unable to detect ULBP4 transcripts in HepG2 cells. Collectively, our data on mAb 6E6 and 709116 advise substantial caution when interpreting results obtained with these commercially available ULBP4 antibodies and strongly suggest to validate such data by independent methodological approaches such as by qPCR and/or immunoblotting. Our data also indicate that in contrast to other NKG2DL such as MICA, MICB, and ULBP1–3, which are frequently expressed on a broad variety of human tumor cell lines (43), ULBP4 may be not or only sparsely expressed on the surface of human tumor cell lines. In fact, immunoblotting of lysates of HeLa cells revealed that the vast majority of detectable ULBP4 glycoproteins is sensitive for digestion with endoglycosidase H, and therefore retained intracellularly in the ER or Golgi complex well in accordance with the lack of detectable cell surface expression (Figures 4C,D). Assessing expression of ULBP4 isoform 1 versus isoform 0 in more than 30 human cell lines, we found that three cell lines including HeLa cells (HeLa, LNCaP, and MG-63) and primary keratinocytes expressed both isoforms, 8 cell lines expressed only isoform 0, but none expressed only isoform 1 (data not shown). Based on these findings, one may consider the possibility that the previously reported isoform 1 rather is a byproduct of inefficient splicing of ULBP4 primary transcripts, whereas isoform 0 may represent the physiologically more relevant isoform. Future research on ULBP4 should take such considerations into account.

### Soluble ULBP4

Isoform 3 may represent a soluble variant of ULBP4 generated by alternative splicing of the full-length isoform 0 excluding exon 4 (Figure 1). However, evidence for isoform 3 thus far relies only on analyses of transcripts/cDNA, but not on protein data (24). Therefore, existence and secretion of sULBP4 based on isoform 3 transcripts has to await validation by experimental evidence. In addition, there are reports claiming the existence of other sULBP4 isoforms generated by alternative splicing (23, 44). However, for the originally reported soluble RAET1E2 isoform (Uniprot isoform 4) (44), no corresponding transcripts could be detected in a subsequent study by the same group (23). This latter study reported three other rarely occurring ULBP4 splice variants all comprising the entire exon 4 encoding for the transmembrane domain. Accordingly, these variants (Uniprot isoforms 2, 5, and 6) reportedly are cell membrane-bound variants (23), which obviously cannot be secreted without further processing. Considering physiologic expression of ULBP4 in skin, we assessed freshly isolated human keratinocytes for ULBP4 transcripts. By pairing a primer located in exon 1 with a reverse primer in the 3′ end of exon 4 (isoform 1) or in the 5′ end of exon 5 (isoform 0), respectively, we detected predominant expression of transcripts corresponding to both full-length isoforms 0 and 1. At considerably lower frequency, we also detected transcripts corresponding to isoforms 2 and 3, but not to isoforms 4–6 (data not shown). Hence, our data indicate, that sULBP4 may be generated physiologically by alternative splicing under exclusion of exon 4 (i.e., ULBP4 isoform 3) although existence of such sULBP4 glycoproteins remains to be shown experimentally. We and others had previously reported that most human NKG2DL can be released from cells either by proteolytic shedding or by exosomal secretion (12, 34, 45–47). MICA (but not MICA*08), MICB, and ULBP2 have been shown to be shed by metalloproteases such as ADAM10, ADAM17, and MMP14 (48–51) while MICA*08 and ULBP3 were shown to be preferentially released in exosomes (45, 52). However, neither proteolytic shedding nor exosomal release has been reported for ULBP4 possibly due to the scarcity of *bona fide* ULBP4-specific antibodies.

**Figure 5:**
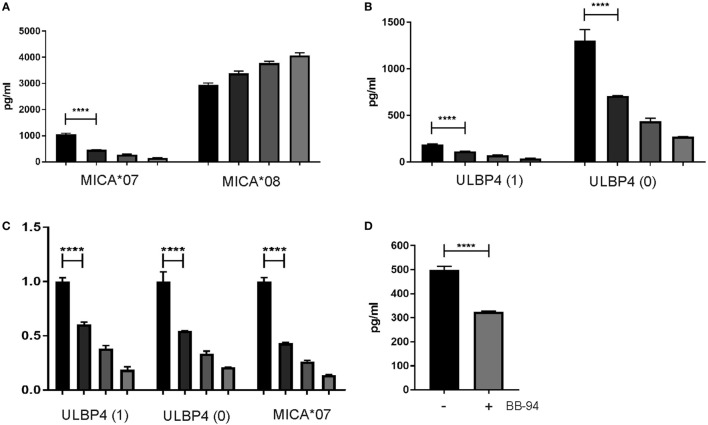
Proteolytic release of soluble ULBP4 (sULBP4). **(A–C)** C1R cells (1 × 10^6^ cells/ml) stably transfected with ULBP4 isoforms 0, ULBP4 isoform 1, MICA*07, or MICA*08, respectively, were cultivated in the presence of various concentrations of batimastat for 42 h (from left to right: 0, 0.2, 1, and 5 µM batimastat). Subsequently, supernatants were analyzed for concentrations of **(A)** sMICA or **(B)** sULBP4. **(C)** Relative changes of concentrations (mean ± SD) as depicted in panels **(A,B)** with values for control-treated samples (0 µM batimastat) set as 1. One representative experiment out of three is shown. Statistical significance was only assessed for differences between samples treated with 0 versus 0.2 µM batimastat using a one-way ANOVA with Tukey’s multiple comparisons test (****p < 0.0001). **(D)** Detection of sULBP4 in supernatants of HeLa cells grown for 65 h in absence or presence of 10 µM batimastat (BB-94). Statistical significance was calculated using unpaired *t*-test (****p < 0.0001).

To address release of sULBP4 from ULBP4-expressing cells, we established a sandwich assay specifically detecting sULBP4 using immobilized anti-ULBP4 pAb and biotinylated mAb DUMO1. Using this assay, sULBP4 was clearly detectable in supernatants of C1R cells stably transfected with ULBP4 isoforms 0 and 1, respectively (Figure 5B). To assess whether metalloproteases are involved in the generation of sULBP4, we added batimastat (BB-94), a potent, broad spectrum metalloprotease inhibitor, to cultures of C1R transfectants. As expected and in line with the previous reports, addition of BB-94 to C1R-MICA*07 and C1R-MICA*08 cells inhibited release of sMICA*07, but not of sMICA*08, in a dose-dependent manner (Figure 5A). Of note, release of sULBP4 from both C1R-ULBP4 transfectants was also inhibited in a BB-94 dose-dependent manner demonstrating that both ULBP4 isoforms 0 and 1 can give rise to sULBP4 due to the action of metalloproteases (Figures 5B,C). Finally, we assessed sULBP4 in the culture supernatants of various cell lines expressing ULBP4 transcripts (Figure 4B). In line with the highest abundance of ULBP4 transcripts in HeLa cells, we only detected low amounts sULBP4 in the supernatants of HeLa cells (Figure 5D), but not of HCT116, HepG2, and HaCat cells (data not shown). Release of sULBP4 by HeLa cells could only partially be blocked by addition of BB-94 (Figure 5D) indicating that HeLa cells generate sULBP4 not only by shedding of full-length isoforms 0 and/or 1 through metalloproteases. Since we detected in HeLa cells both transcripts of ULBP4 isoform 3 (data not shown) and ULBP4 proteins of a molecular mass corresponding to isoform 3 (Figure 4C), secretion of isoform 3 may explain the BB-94 independent release of sULBP4 by HeLa cells.

## Concluding Remarks

ULBP4 glycoproteins are among the least characterized human NKG2D ligands. Our present study suggests that this may be explained by a highly restricted expression in tissues and by cell lines as well as by the scarcity and deficiency of commercially available antibodies. While prevalence of ULBP4 transcripts indicates a strong expression bias toward tissues and cell lines of ectodermal origin, physiologic and cellular expression of ULBP4 glycoproteins by such cells remains to be addressed by future research. We here provide evidence that the aminoterminus of mature ULBP4 molecules is recessed when compared with other ULBP molecules and that also the carboxyterminus of at least a substantial portion of ULBP4 molecules substantially differs from the originally reported sequence due to previously unrecognized alternative splicing, resulting in a shortened mature ULBP4 polypeptide which we termed isoform 0. It remains to be shown which isoform is more physiological relevant. Our results also suggest that some previous results obtained with mAb 6E6 and mAb 709116 should be considered with caution. Furthermore, we here demonstrate that ectopically expressed ULBP4 is shed from the cell surface by metalloproteases giving rise to sULBP4 in a manner similarly to many other human NKG2DL. However, in HeLa cells release of sULBP4 was only in part dependent on metalloprotease activity, and possibly a major portion of sULBP4 secreted by HeLa cells is due to the alternatively spliced isoform 3. Of note, most ULBP4 glycoproteins were intracellularly retained in HeLa cells as evident from their lack of EndoH resistance and lack of surface expression, and it remains to be determined by future studies whether this is a peculiar feature of HeLa cells or a general property of ULBP4. In addition, it will be of considerable interest to determine occurrence of sULBP4, either proteolytically shed or as secreted isoform 3, in healthy or diseased state such as in patients with skin or cervical tumors. Altogether, we propose that ULBP4 should not regarded as just another NKG2DL, but that the unique cellular expression and processing of ULBP4 together with the early evolutionary separation from other ULBP family members indicates a peculiar function within the framework of NKG2D-mediated immunosurveillance.

## Ethics Statement

Study includes analyses of buffy coats obtained from the Blood Donor Service Baden-Wuerttemberg-Hessen, Frankfurt am Main, after approval by the Ethics committee of the Goethe University of Frankfurt. Study includes generation of monoclonal antibodies based on immunization of mice which was carried out in accordance with current laws for animal research and approved by the Regierungspräsidium Tübingen and Darmstadt.

## Author Contributions

TZ, MH, and MW designed and performed experiments and analyzed data. AS conceptualized study, designed experiments, and wrote the manuscript with the support of TZ and MH.

## Conflict of Interest Statement

The authors declare that the research was conducted in the absence of any commercial or financial relationships that could be construed as a potential conflict of interest.

## Footnotes

^1^http://www.uniprot.org/uniprot/Q8TD07 (Accessed: January 31, 2018).

^2^https://www.scbt.com/scbt/product/ulbp4-antibody-6e6 (Accessed: January 31, 2018).

^3^https://www.proteinatlas.org/ENSG00000164520-RAET1E/tissue(Accessed: January 31, 2018).

^4^https://www.proteinatlas.org/ENSG00000164520-RAET1E/cell (Accessed: January 31, 2018).

^5^https://www.rndsystems.com/products/human-ulbp-4-raet1e-apc-conjugated-antibody-709116_fab6285a (Accessed: January 31, 2018).
